# Increased Plasma Clot Permeability and Susceptibility to Lysis Are Associated with Heavy Menstrual Bleeding of Unknown Cause: A Case-Control Study

**DOI:** 10.1371/journal.pone.0125069

**Published:** 2015-04-24

**Authors:** Piotr Szczepaniak, Michał Zabczyk, Anetta Undas

**Affiliations:** 1 The John Paul II Hospital, Krakow, Poland; 2 Institute of Cardiology, Jagiellonian University Medical College, Krakow, Poland; IIBB-CSIC-IDIBAPS, SPAIN

## Abstract

**Background:**

Formation of compact and poorly lysable clots has been reported in thromboembolic disorders. Little is known about clot properties in bleeding disorders.

**Objectives:**

We hypothesized that more permeable and lysis-sensitive fibrin clots can be detected in women with heavy menstrual bleeding (HMB).

**Methods:**

We studied 52 women with HMB of unknown cause and 52 age-matched control women. Plasma clot permeability (K_s_), turbidity and efficiency of fibrinolysis, together with coagulation factors, fibrinolysis proteins, and platelet aggregation were measured.

**Results:**

Women with HMB formed looser plasma fibrin clots (+16% [95%CI 7–18%] Ks) that displayed lower maximum absorbancy (-7% [95%CI -9 – -1%] ΔAbs_max_), and shorter clot lysis time (-17% [95%CI -23 – -11%] CLT). The HMB patients and controls did not differ with regard to coagulation factors, fibrinogen, von Willebrand antigen, thrombin generation markers and the proportion of subjects with defective platelet aggregation. The patients had lower platelet count (-12% [95%CI -19 – -2%]), tissue plasminogen activator antigen (-39% [95%CI -41 – -29%] tPA:Ag), and plasminogen activator inhibitor-1 antigen (-28% [95%CI -38 – -18%] PAI-1:Ag) compared with the controls. Multiple regression analysis upon adjustment for age, body mass index, glucose, and fibrinogen showed that decreased tPA:Ag and shortened CLT were the independent predictors of HMB.

**Conclusions:**

Increased clot permeability and susceptibility to fibrinolysis are associated with HMB, suggesting that altered plasma fibrin clot properties might contribute to bleeding disorders of unknown origin.

## Introduction

Heavy menstrual bleeding (HMB), that occurs at normal intervals (21–35 days) but lasts longer than 7 days or is associated with blood loss of more than 80 ml, affects 5% of the population of women mostly in reproductive age [1–2]. Over a half of the patients with HMB do not manifest any organic pathology. HMB is responsible for iron deficiency in women and lowers their quality of life [3]. A number of known causes of HMB have been identified, including several hemostatic disorders, particularly von Willebrand disease (VWD) that occurs in 5–20% of patients with HMB as compared with less than 1% of women with normal menstruation [4]. Other known pathologies underlying HMB are: inherited bleeding disorders (e.g. haemophilia), endometrial polyps or carcinoma, use of contraceptive pills or hormonal therapy, diet changes, treatment with anticoagulants, antiplatelet agents or certain drugs (e.g. steroids), and gestational complications (e.g. miscarriages) [5–7]. The first study of haemostasis in patients with HMB was published about 20 years ago [8], however little is known about clot properties in HMB.

There is growing evidence that plasma fibrin clots composed of compact networks with thin fibers that are less susceptible to lysis [9] can be observed in patients with a history of myocardial infarction [10], ischemic stroke [11], and peripheral artery disease [12]. It is unclear whether specific abnormalities in plasma fibrin clot properties can be detected in patients with bleeding tendency. Haemophilia A and B have been shown to be characterized by delayed and slower production of fibrin with the subsequent formation of plasma fibrin clots composed of thick fibrin fibres with larger pores that are more susceptible to lysis [13]. These abnormalities have been postulated to be largely caused by reduced thrombin generation in haemophilia [13]. Furthermore, some congenital dysfibrinogenemias could be associated with HMB, e.g. in the fibrinogen AαIVS4+1G>T heterozygous mutation with the Aα4841delC mutation in the other allele [14].

Plasma fibrin clot formation and structural characteristics in subjects with HMB of unknown origin have not been reported yet. We hypothesized that such women with HMB have abnormal plasma fibrin clot properties including increased clot permeability and lysability. We sought to investigate plasma clot characteristics in HMB and their potential determinants in a case-control study.

## Materials and Methods

### Patients

Of the 130 screened women, we examined 52 consecutive women aged 50 years or less diagnosed with HMB who were referred by gynecologists to the Center for Coagulation Disorders for further laboratory work-up between January 2009 and June 2012. Fifty-two age-and weight-matched (by frequency) apparently healthy women with a subjectively normal menstruation, recruited among the hospital personnel and relatives (n = 88), served as controls. The 36 healthy control women were excluded from the study due to the use of progestogens or combined oral contraceptives, intrauterine devices or other medications, in particular aspirin or other nonsteroidal anti-inflammatory agents. All patients with HMB had a history of heavy, regular menstrual periods, defined on the basis of the scoring system of Higham et al. [15]. The exclusion criteria were uterine pathologies including polyps, fibroid tumours, endometritis; known hematologic disorders, including VWD and deficiencies of coagulation factors; the use of nonsteroidal anti-inflammatory agents (in particular aspirin), antifibrinolytics or anticoagulants; platelet count below 100 G/l; sideropenic anemia; acute infections or severe comorbidities (e.g. malignancy, renal insufficiency). Only women who did not take progestogens or combined oral contraceptives and could not use of intrauterine devices in the previous 3 months were eligible. All the individuals declared not taking any medications within the 2 weeks prior to enrollment. Iron replacement therapy within the previous 6 months was reported by 28 women. We recorded bleeding episodes based on medical records and self-declared signs and symptoms such as epistaxis, easy bruising, gingival bleeding and gastrointestinal bleeding in the past. Family history of bleeding was arbitrarily defined as self-reported easy bruising or other bleeding tendency in any family member including first and second degree relatives. The Bioethics Committee of the Jagiellonian University approved the study. All the subjects signed written informed consent.

### Laboratory investigations

Fasting blood was drawn from an antecubital vein with minimal stasis within the first 10 days after menstruation. Plasma samples (9:1 of 3.2% trisodium citrate) for fibrin clot assays were centrifuged (20 min, 2500 g) within 30 minutes of collection to obtain platelet-poor plasma, immediately frozen, and stored in aliquots at—80°C. Lipid profiles, blood cell counts, glucose, creatinine, D-dimer, international normalized ratio (INR), and activated partial thromboplastin time (APTT) were assayed by routine laboratory techniques. Serum ferritin was measured by the latex-enhanced immunoturbidimetry (Roche Diagnostics, Mannheim, Germany). Fibrinogen was determined using the von Clauss method. High-sensitivity C-reactive protein (CRP) was measured by latex nephelometry (Siemens). Factor (F)II, FV, FVII, FVIII, FIX, and FX were measured by one-stage clotting assays using factor-deficient plasma (Siemens, Marburg, Germany). FXIII activity was determined by the chromogenic assay (Siemens, Marburg, Germany). All patients had coagulation factors above 70% of the reference range. VWF antigen (vWF:Ag) was measured by latex immunoassay on a STAR coagulation instrument (Diagnostica Stago, Asnieres, France). All patients had vWF:Ag above 50 IU/dL. Tissue-type plasminogen activator (tPA), plasminogen activator inhibitor-1 (PAI-1) antigens and plasmin-alpha 2-antiplasmin complexes (PAP) were measured by enzyme-linked immunoabsorbent assays (ELISAs) (all, American Diagnostica, Stamford, CT). The interassay and intraassay coefficients of variation for the ELISAs were <8%.

### Platelet aggregation

Platelet function was assessed by means of aggregometry as described [16]. The citrated blood samples were centrifuged at 120 g for 10 min for platelet-rich plasma and then at 850 g for 10 min for platelet-poor plasma. Platelet reactivity was evaluated in platelet-rich plasma for 6 minutes at 37°C using the light transmittance aggregometer (Chrono-Log Corp., Haverton, PA) after stimulation with 5 μM adenosine diphosphate (ADP). Maximal platelet aggregation after stimulation was expressed as the maximal percent change in light transmittance in platelet-rich plasma from baseline, using platelet-poor plasma as a reference. Defective aggregation was defined as a maximal values below 75% of the reference values.

### Clot permeability

Permeation of plasma fibrin clots was determined as described [17–19]. Plasma was diluted 1:1. Briefly, 20 mM calcium chloride and 1 U/mL human thrombin (Sigma) were added to citrated plasma. Tubes containing the clots were connected to a reservoir of a Tris-buffered saline (TBS) buffer (0.01 M Tris, 0.1 M NaCl, pH 7.5), and its volume flowing through the gels was measured within 60 minutes. A permeation coefficient (K_s_), which indicates the pore size, was calculated from the equation: K_s_ = QxLxη/txAxΔp, where Q is the flow rate in time t, L is the length of a fibrin gel, η is the viscosity of liquid (in poise), t is percolating time, A is the cross-sectional area (in cm^2^), and Δp is a differential pressure (in dyne/cm^2^). The interassay variability was 7.4%.

### Turbidity measurements

Plasma samples were diluted 1:1 with a buffer (0.05 M Tris-HCl, 0.15 M NaCl, pH 7.4) containing 1 U/mL human thrombin (Sigma) and 15 mM calcium chloride to start fibrin polymerization [16,17]. Absorbance was read at 405 nm for 15 minutes with a Perkin-Elmer Lambda 4B spectrophotometer (Molecular Devices Corp). The lag phase of the turbidity curve is the time required for fibrin protofibrils to grow to sufficient length to allow lateral aggregation to occur. Maximum absorbance at plateau (ΔAbs_max_) reflects the number of protofibrils per fiber. The interassay and intraassay coefficients of variation were <7%.

### Clot lysis assays

Assay 1. In the first assay, clot lysis time (CLT) was measured using the turbidity method of Lisman et al. with a slight modification [19] Briefly, plasma was mixed with 15 mM calcium chloride, 10000-diluted human 0.6 pM tissue factor (Innovin, Dade Behring), 12 μM phospholipid vesicles and 60 ng/mL recombinant tPA (Boehringer Ingelheim, Germany). Measurements were performed at 405 nm at 37 °C. Clot lysis time was defined as the time from the midpoint of the clear-to-maximum-turbid transition, which represents clot formation, to the midpoint of the maximum-turbid-to-clear transition.

Assay 2. Plasma fibrin clots formed as described in the permeability assay were perfused with the same buffer containing 0.2 μM recombinant tPA [18,20]. The lysis rate was determined by measuring the concentration of D-dimer (American Diagnostica), a marker of plasmin-mediated fibrin degradation, every 15 minutes in the effluent. Maximum rates of increase in D-dimer levels (D-D_rate_, mg/L/min) was analyzed in each subject. The experiment was stopped when the fibrin gel collapsed. The interassay coefficients of variation for lysis variables were <7%.

### Thrombin Generation Potential

To assess plasma thrombogenic potential, the thrombogram was analyzed using the CAT (Thrombinoscope BV, Maastricht, the Netherlands) according to the manufacturer’s instructions in the 96-well plate fluorometer (Ascent Reader, Thermolabsystems OY, Helsinki, Finland) equipped with the 390/460 filter set at a temperature of 37°C. Eighty microliters of platelet-poor plasma was diluted with 20 μL of the reagent containing 5 pmol/L recombinant tissue factor, 4 micromolar phosphatidylserine/phosphatidylcholine/phosphatidylethanolamine vesicles, and 20 μL of FluCa solution (Hepes, pH 7.35, 100 nmol/L CaCl2, 60 mg/mL bovine albumin, and 2.5 mmol/L Z-Gly-Gly-Arg-amido methyl coumarin). Each plasma sample was analyzed in duplicate, and the intraassay variability was 6%. For analysis, the maximum concentration of thrombin generated and Endogenous Thrombin Potential (ETP) were used.

### Scanning electron microscopy (SEM)

Plasma fibrin clots from randomly selected patients and controls were analyzed. Fixation was performed after the permeability measurement with the use of 2.5% of glutaraldehyde in phosphate-buffered saline solution for 2 hours. Fixed clots were gently removed from tubes, washed with distilled water, and then dehydrated in graded water-ethanol solutions, dried by the critical point procedure, and sputter coated with gold. Samples were scanned in six different areas (microscope JEOL JCM-6000; JEOL Ltd., Tokyo, Japan).

### Statistical analysis

The data are shown as median (range), mean ± standard deviation (SD), or percentage as appropriate. Continuous variables were first checked for normal distribution by the Shapiro-Wilk test. The Mann-Whitney U or Student tests were used as appropriate. The χ^2^ test was used to compare the category frequencies. The Pearson or Spearman rank correlation coefficients were calculated to test the association between 2 variables with a normal or non-normal distribution, respectively. The relative risk of HMB associated with K_s_, CLT and lag phase was estimated as an odds ratio (OR) and corresponding 95% confidence interval (CI) using logistic regression, adjusted for age, body mass index (BMI), glucose, and fibrinogen. Percentiles (70th, 80th, 90th, and 95th) of the K_s,_ and CLT measured in the control group were used as the cut-off levels. All clinical and angiographic variables that showed the association with HMB were then included in the stepwise multiple logistic regression analysis. *P*< 0.05 was considered statistically significant. Variables were standardized to age, BMI, fibrinogen and glucose. Independent predictors of HMB were evaluated using Receiver Operating Curve (ROC) analysis. The cut-off values were calculated by using the Youden index.

The study was powered to have a 90% chance of detecting a 10% difference in CLT using a *P* value of 0.01, based on the values of CLT in the published article [21]. In order to demonstrate such a difference or greater, 32 patients were required in each group.

## Results

The patient and control groups were matched for age, BMI, and the prevalence of current smoking (Table 1). Bleeding tendency manifestations, including easy bruising, were self-reported in similar proportions of the subjects in both groups (Table 1). Women with HMB had normal, but slightly lower platelet count (by 12%) with similar proportions of subjects with defective platelet aggregation compared with the controls (Table 1). Ferritin, APTT and vWF:Ag levels did not differ between the groups (Table 1). Coagulation factors, including fibrinogen, as well as routine coagulation tests and D-dimer were similar in both groups (Tables 1 and 2). Of note, patients with HMB had lower tPA:Ag and PAI-1:Ag with a positive association (r = 0.52; *P =* 0.01) between these two variables (Table 3). Women with HMB had 7.5% higher PAP compared with the controls (Table 1). ETP and the maximum concentration of thrombin generated were similar in both groups (Table 3).

**Table 1:** Characteristics of patients with heavy menstrual bleeding and controls.

Variables	Patients (n = 52)	Control subjects (n = 52)	*P* value
Age, y^a^	36 (29–44)	39 (30–45)	0.49
BMI, kg/m^2^ ^a^	25.7 (24.0–29.5)	25.1 (23.8–27.4)	0.30
Length of cycle, d^a^	27 (24–31)	26 (24–29)	0.38
Duration of period, d^a^	9 (8–12)	6 (4–7)	< 0.001
Hemoglobin, g/dL^a^	13.4 (12.4–14.3)	13.9 (12.7–14.7)	0.23
Platelets, x10^3^/μL^a^	219.5 (196.5–264.5)	249.5 (205.0–290.0)	0.02
Total cholesterol, mM^a^	5.20 (4.23–5.77)	4.93 (4.22–5.62)	0.55
LDL cholesterol, mM^a^	2.97 (2.36–3.33)	2.99 (2.50–3.50)	0.65
HDL cholesterol, mM^a^	1.54 (1.16–1.72)	1.39 (1.13–1.72)	0.46
Triglycerides, mM^a^	1.17 (0.67–1.58)	1.02 (0.70–1.53)	0.89
Glucose, mM^a^	4.9 (4.5–5.1)	4.6 (4.3–5.1)	0.12
Creatinine, μM^b^	67.9 ± 15.6	68.4 ± 8.4	0.86
hsCRP, mg/L^a^	1.35 (0.80–2.22)	1.77 (1.01–2.39)	0.77
INR^a^	1.00 (0.92–1.06)	0.98 (0.91–1.05)	0.57
APTT, s^a^	28.7 (26.9–30.9)	28.9 (27.4–30.9)	0.79
Fibrinogen, g/L^a^	2.5 (2.3–2.7)	2.6 (2.3–3.2)	0.26
D-dimer, mg/dL^b^	247 ± 10	256 ± 14	0.58
tPA:Ag, ng/mL^a^	5.96 (5.11–7.02)	9.72 (8.65–10.88)	< 0.001
PAI-1:Ag, ng/mL^b^	8.54 ± 1.85	11.89 ± 3.84	< 0.001
PAP, μg/L^a^	232.0 (202.0–283.5)	214.5 (167.5–257.0)	0.04
Ferritin, ng/mL^a^	68.8 (17.2–115.2)	47.3 (18.9–146.6)	0.40
vWF:Ag, IU/dL^a^	99.0 (89.0–108.0)	99.5 (91.5–108.5)	0.70
Age at onset of heavy menstrual bleeding, y^a^	18 (16–26)	-	-
Current smokers, n (%)^c^	12 (23)	12 (23)	1.00
Family history, n (%)^c^	8 (15)	11 (21)	0.45
Bruising, n (%)^c^	11 (21)	9 (17)	0.61
Epistaxis, n (%)^c^	6 (12)	3 (6)	0.29
Severe bleeds, n (%)^c^	2 (4)	0 (0)	0.15
Defective aggregation, n (%)^c^	12 (23)	16 (31)	0.38

BMI, body mass index; LDL, low-density lipoprotein; HDL, high-density lipoprotein; hsCRP, high-sensitivity C-reactive protein; INR, international normalized ratio; APTT, activated partial thromboplastin time; tPA:Ag, tissue plasminogen activator antigen; PAI- 1:Ag, plasminogen activator inhibitor-1 antigen; PAP, plasmin-alpha 2-antiplasmin complexes; vWF:Ag, von Willebrand factor antigen.

^a^Data are shown as median (interquartile range); Comparison between 2 groups using the Mann Whitney U test;

^b^Data are shown as mean ± standard deviation; Comparison between 2 groups using the Student t-test;

^c^Comparison between 2 groups using χ^2^ test.

**Table 2:** Coagulation factors in patients with heavy menstrual bleeding and controls.

Coagulation factor	Patients (n = 52)	Control subjects (n = 52)	*P* value
FII (IU/dL)	103.8 (94.3–117.1)	107.2 (93.2–122.1)	0.37
FV (IU/dL)	100.5 (86.5–109.5)	96.4 (83.3–105.6)	0.21
FVII (IU/dL)	101.0 (95.6–108.2)	103.2 (92.4–111.1)	0.94
FVIII (IU/dL)	113.3 (88.7–131.6)	116.7 (101.5–128.7)	0.43
FIX (IU/dL)	102.2 (95.0–112.0)	100.0 (89.0–108.1)	0.10
FX (IU/dL)	101.5 (94.7–111.0)	99.1 (87.8–111.7)	0.37
FXI (IU/dL)	101.5 (94.0–110.0)	100.2 (86.1–111.4)	0.26
FXIII (%)	101.3 ± 10.0	101.0 ± 9.2	0.82

F, factor.

Data are shown as median (interquartile range) or mean ± standard deviation (for FXIII); Comparison between 2 groups using Mann Whitney U test or Student t-test.

**Table 3:** Comparisons of fibrin clot variables and thrombin generation parameters in patients with heavy menstrual bleeding and controls.

Variables	Patients (n = 52)	Control subjects (n = 52)	*P* value
K_s_ (10^–9^ cm^2^) ^a^	8.9 (7.8–9.4)	7.5 (7.0–8.1)	< 0.001
Lag phase (s) ^a^	45 (42–49)	43 (40–46)	0.05
ΔAbs_max_ (405 nm) ^a^	0.75 (0.73–0.84)	0.81 (0.77–0.87)	0.02
CLT (min) ^b^	66.5 ± 11.9	80.6 ± 12.7	< 0.001
D-D_rate_ (mg/L/min) ^a^	0.072 (0.070–0.077)	0.072 (0.068–0.079)	0.60
ETP (nM x min) ^a^	1241 (1185–1368)	1290 (1148–1437)	0.40
Peak thrombin generation (nM) ^a^	206 (169–272)	239 (198–271)	0.24

K_s_, permeability coefficient; ΔAbs_max_ (405 nm), maximum absorbancy of a fibrin gel at 405 nm; CLT, clot lysis time; D-D_rate_, maximum rate of increase in D-dimer levels; ETP, endogenous thrombin potential; Peak thrombin generation, maximum concentration of thrombin generated.

^a^Data are shown as median (interquartile range); Comparison of subjects between 2 groups with analysis of Mann Whitney U test;

^b^Data are shown as mean ± standard deviation; Comparison of subjects between 2 groups with analysis of Student test.

### Clot permeability

Plasma fibrin clots from the HMB patients were more permeable than those from the controls (Table 3). K_s_ was associated with fibrinogen (r = -0.52), D-dimer (r = -0.30), APTT (r = 0.33), and tPA:Ag (r = -0.39; all *P*< 0.05). There were no correlations between K_s_ and duration of the period or the presence of other bleeding manifestations (data not shown). Multiple regression analysis adjusted for age, BMI, fibrinogen, and glucose showed that K_s_ was independently associated with the length of a cycle alone (*R* ^*2*^ = 0.49, *P =* 0.02; β = 0.32, 95% CI: 0.29–0.35) in the HMB patients. To investigate the potential contribution of K_s_ to the presence of HMB, different cut-off levels were set according to the 70th, 80th, 90th, and 95th percentiles (Table 4).

**Table 4:** Risk of heavy menstrual bleeding according to permeability coefficient (K_s_).

Cut-off percentile	Cut-off K_s_, 10^–9^ cm^2^	No. of controls	No. of cases	OR (95% CI) ^a^	*P* value
70	8.0	8	29	9.27 (3.16–27.2)	< 0.001
80	8.3	7	21	4.73 (1.62–13.9)	0.005
90	9.5	6	12	2.02 (0.64–6.41)	0.232
95	9.6	4	9	2.21 (0.60–8.13)	0.234

^a^ Odds ratios (ORs) adjusted for age, BMI, glucose and fibrinogen; CI, confidence interval.

### Turbidity measurements

Compared with control subjects, HMB patients had slightly altered turbidimetric curves (Table 3). The lag phase was correlated with age at the onset of HMB (r = 0.33), duration of the period (r = 0.40), and tPA:Ag (r = -0.44; all *P<* 0.05). In the patient group ΔAbs_max_ correlated with fibrinogen (r = 0.43; *P<* 0.05). Multiple regression analysis adjusted for age, BMI, fibrinogen, and glucose showed that the lag phase was independently associated with age at the onset of HMB (*R* ^*2*^ = 0.45, *P =* 0.01; β = 0.54, 95% CI: 0.49–0.58), while we identified no independent predictors of ΔAbs_max_.

### Clot lysis

CLT was shorter in women with HMB compared with controls (Table 3). In these patients, CLT was inversely correlated with hemoglobin (r = -0.36), PAP (r = -0.50) and positively associated with fibrinogen (r = 0.35), and FX (r = 0.34; all *P<* 0.05), but not with PAI-1:Ag. There were also no associations with CLT and either the length of the cycle or duration of the period (data not shown). Multiple regression analysis adjusted for age, BMI, fibrinogen, and glucose showed that CLT was independently associated only with tPA:Ag (*R* ^*2*^ = 0.41, *P =* 0.014; β = 0.24, 95% CI: 0.21–0.27) in the HMB patients. To investigate the potential contribution of CLT to the presence of HMB, different cut-off levels were set, according to the 70th, 80th, 90th, and 95th percentiles (Table 5). Time-courses of the D-dimer release from plasma fibrin clots demonstrated that D-D_rate_ in this assay did not differ between the groups (Table 3). D-D_rate_ was correlated with duration of the period (r = -0.35), and D-dimer (r = 0.36; all *P*< 0.05). Multiple regression analysis adjusted for age, BMI, fibrinogen, and glucose showed that the D-D_rate_ was independently associated only with VWF:Ag (*R* ^*2*^ = 0.45, *P =* 0.02; β = 0.39, 95% CI: 0.34–0.43) and FXI (*R* ^*2*^ = 0.44, *P =* 0.04; β = -0.39, 95% CI: −0.44- −0.34), respectively, in the HMB patients.

**Table 5:** Risk of heavy menstrual bleeding according to clot lysis time (CLT).

Cut-off percentile	Cut-off CLT, min	No. of controls	No. of cases	OR (95% CI) ^a^	*P* value
70	89	27	10	0.22 (0.09–0.54)	0.001
80	95	21	7	0.23 (0.08–0.63)	0.004
90	99	15	3	0.15 (0.03–0.54)	0.005
95	100	12	1	0.05 (0.01–0.45)	0.008

^a^ ORs adjusted for age, BMI, glucose and fibrinogen. Abbreviations: see Table 4.

### Regression and ROC analysis

**Fig 1:**
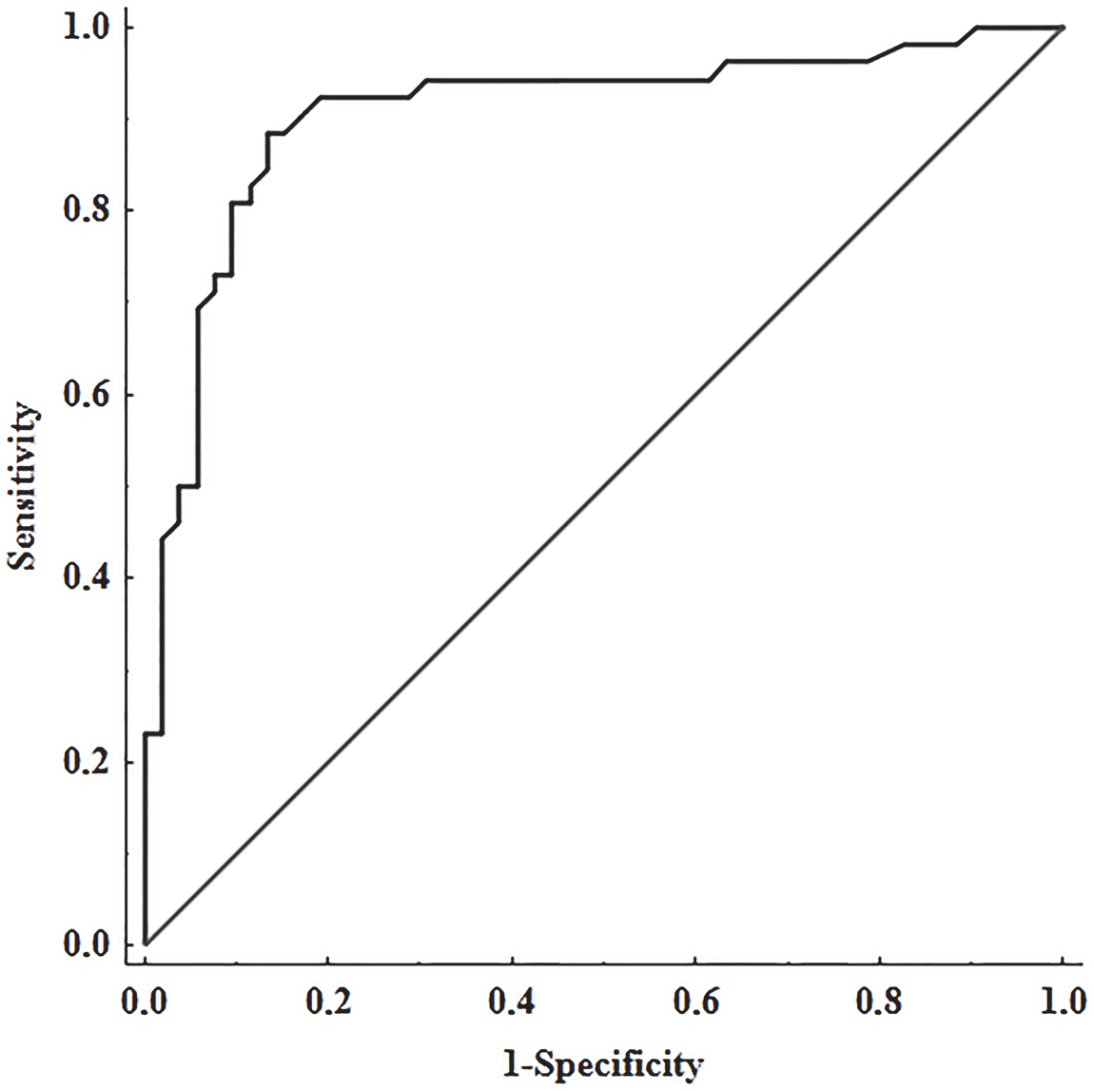
ROC curve for tissue plasminogen activator antigen (tPA:Ag). AUC denotes area under curve; SE, standard error; CI, confidence intervals.

**Fig 2:**
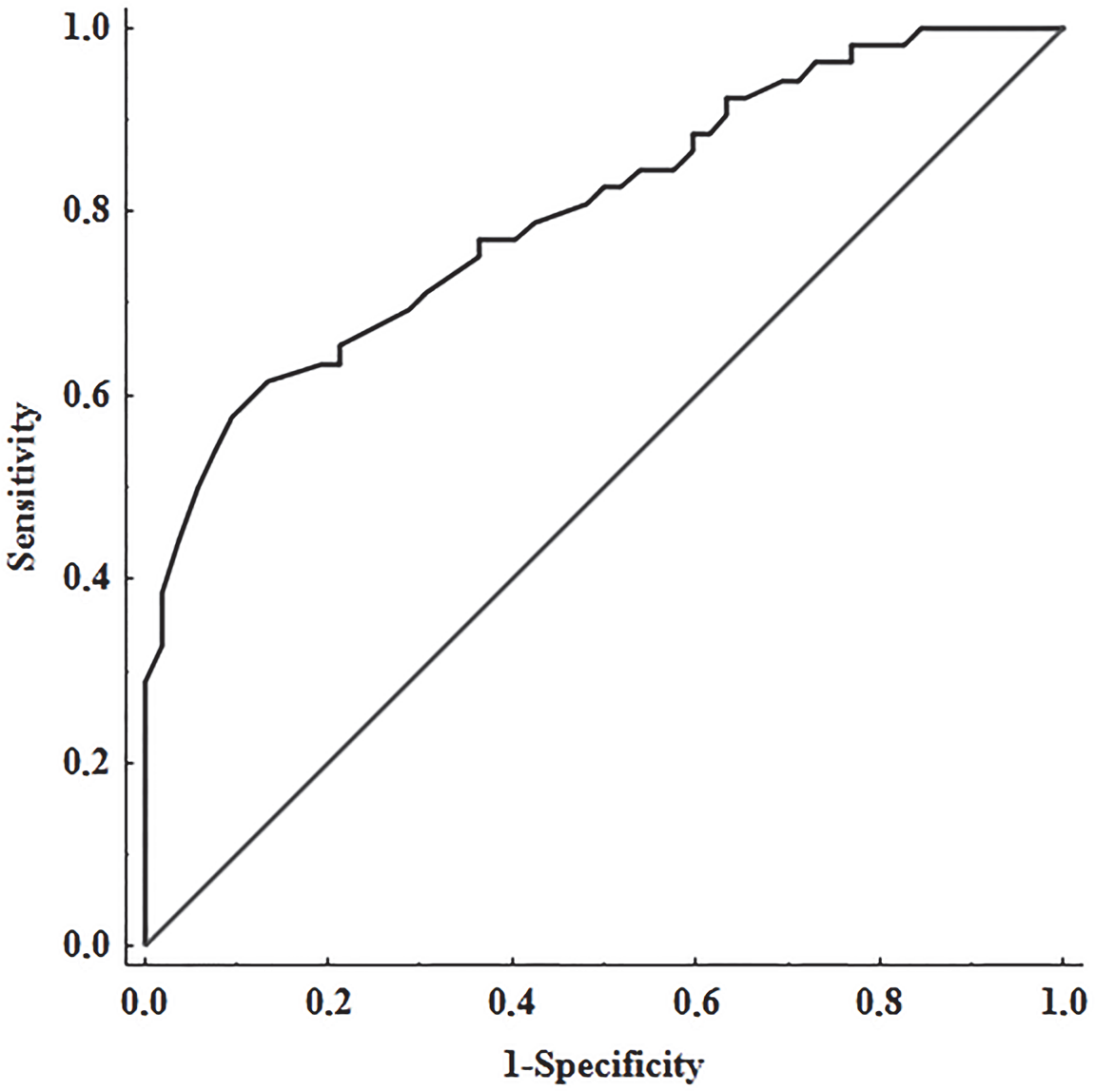
ROC curve for clot lysis time (CLT). AUC denotes area under curve; SE, standard error; CI, confidence intervals.

Variables such as age, BMI, glucose, and fibrinogen were used for standardization. Multivariate model of stepwise multiple logistic regression analysis, including all the subjects studied (n = 104), showed that tPA:Ag and CLT were the only independent predictors of HMB. After adjusting for age, BMI, glucose, and fibrinogen, ORs were 0.38 (95% CI 0.26–0.56; *P<* 0.001) for tPA:Ag (per 1 ng/mL) and 0.93 (95% CI 0.88–0.98; *P =* 0.014) for CLT (per 1 min). As shown in Figs 1 and 2, the highest area under the curve (AUC) was found for tPA:Ag (AUC = 0.906, 95%CI = 0.843–0.968, cut-off = 7.74 ng/mL, sensitivity = 0.885, specificity = 0.875), while AUC for CLT was 0.800 (95%CI = 0.717–0.884, cut-off = 66 min, sensitivity = 0.615, specificity = 0.865).

### Clot structure

**Fig 3:**
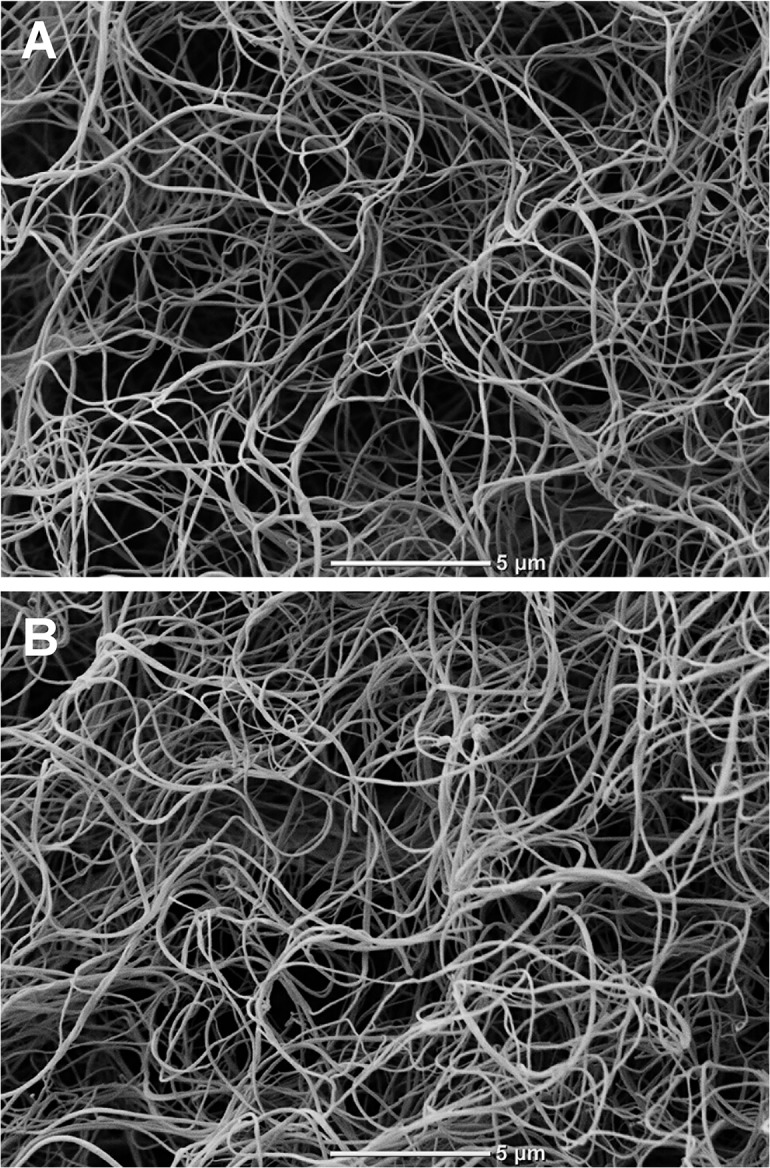
Representative SEM images of plasma fibrin clots. (A) a patient with heavy menstrual bleeding (HMB). (B) a healthy control subject. Magnification 5000x. Scale bar 5 μm.

Representative SEM images (Fig 3) showed that the plasma clots from the HMB patients were composed of more loosely packed fibrin fibres than those from the healthy controls.

## Discussion

The current study demonstrates that abnormal plasma fibrin clot properties, including formation of less compact fibrin networks displaying faster lysability, occur in women with heavy prolonged menstrual bleeding which cause cannot be identified. To our knowledge, this study is the first to show that slightly, though significantly increased plasma clot permeability in women with HMB. Our findings show that abnormal structure and function of fibrin network are involved not only in thrombotic disorders [9–12], but also in such common bleeding disorders as HMB in women free of known deficiencies in coagulation factors, thrombocytopenia, VWD or other hemorrhagic diatheses. Since the proper balance between fibrin formation and degradation is necessary to protect the vascular system from excess blood loss, our findings indicate that formation of looser clots sensitive to tPA-mediated lysis might contribute to HMB.

Very recently, Wiewel-Verschueren et al. [22] have compared clot lysis time in 102 women with HMB versus 28 healthy controls, and they did not observe lower values in the former group. The reasons for the discrepant results of that and our study are likely associated with different patient characteristics and different selection methods of patients. In contrast to the our study, Wiewel-Verschueren et al. [22] did not exclude women with gynaecological abnormalities (present in 33% of the patients, no data for controls) and studied also female patients mostly above 40 years of age, with low haemoglobin and ferritin.

We analyzed several factors that might be implicated in unfavorable plasma fibrin clot features in women with HMB. Since fibrinogen, a key modulator in fibrin properties [9,23], was similar in both groups, the intergroup differences in plasma fibrin clot parameters cannot be explained by any differences in the fibrinogen concentration. A modulatory role of factors II, V,VII, IX, X and XI, which plasma levels were similar and within the reference ranges in the patients and controls, is of negligible importance as a contributor to the intergroup differences in fibrin clot variables in this case-control study. Thrombin generation as well as coagulation factors did not differ between the groups. An inverse correlation of clot permeability with tPA:Ag, which we have reported here, has been observed in previous studies done in subjects without bleeding tendency [9]. Interestingly, a decreased tPA:Ag level, which represents free tPA and tPA bound to PAI-1 [24], was an independent predictor of CLT in women with HMB, which is a novel finding of this study, highlighting a role of this parameter in hemostasis in subjects with bleeding tendency. Moreover, we demonstrated that patients with HMB had slightly, but significantly decreased levels of PAI-1:Ag. A very low activity of PAI-1 indicating deficiency of this inhibitor was shown in case reports describing women with HMB [25]. Slightly lower PAI-1:Ag might facilitate clot lysis by increasing an active free fraction of tPA in the circulation [24]. Positive correlations between CLT and PAI-1:Ag were reported in healthy subjects or those with thrombosis or advanced atherosclerosis [21,26], as well as in the study by Wiewel-Verschuren et al. [22]. We did not observe such association in our patients with HMB. Decreased levels of tPA:Ag and PAI-1:Ag have already been shown to occur in young women with a high fibrinolytic potential [27]. Since decreased levels of tPA:Ag and PAI-1:Ag were also associated with enhanced fibrinolytic activity in our HMB patients as evidenced by shorter CLT, it might be hypothesized that the net effect of this tPA/PAI-1 pattern leads to reduced inhibition of fibrinolysis most likely indicating a potential involvement of endothelial cell dysfunction as the endothelium is a major source of PAI-1 and tPA [24]. Plasma PAP levels, a sensitive marker of *in vivo* fibrinolysis, were higher in patients than in the controls, which provides additional evidence that a hyperfibrinolytic state occurs in the HMB patients.

Regarding routine hemostatic parameters, Knol et al. [28] observed prolonged APTT in patients with HMB in comparison to the control group. However, we did not show a similar difference and any alterations in coagulation proteins that determine APTT values. Further studies are needed to elucidate whether longer APTT occur in women with HMB without gynaecological abnormalities and known coagulation disorders.

In the context of clot phenotype, of interest is our observation that patients with HMB presented with a normal, but lower platelet count compared with the well-matched control group. Several studies demonstrated that platelet activation, reflected by the release a number of substances, including proteins e.g. platelet factor 4, has been shown to render plasma fibrin clot properties abnormal with formation of denser networks resistant to enzymatic degradation [9,17]. Polyphosphate, a polymer of inorganic phosphate secreted by dense granules of activated platelets, has been demonstrated to unfavorably alter plasma fibrin clot properties, including plasmin-mediated degradation [29]. Analysis of platelet derived proteins that might affect plasma clot phenotype may increase our knowledge on the links between platelets and fibrin properties in patients with HMB. From the methodological point of view, assessment of clot lysis was of particular importance and was performed by means of two assays with the use of various concentrations of tPA. Given differences in response to tPA while using various assays to assess clot lysis, reported in several studies [11–12, 17], we provided evidence that CLT with a relatively low final tPA concentration is the preferred approach that may show a subtle modulation of the fibrinolytic system in women with HMB of unknown cause. At high tPA concentrations, we observed no intergroup differences in fibrinolysis markers such as the rate of increases in D-dimer released from plasma clots. We confirmed that assays for measuring fibrinolysis efficiency should be adjusted to a given disease state and one assay is unlikely to be a good approach for all clinical situations related to hemostatic disorders. The current study had several limitations. First of all, the number of the women studied was relatively low, though the study was sufficiently powered to show intergroup differences. Second, all parameters were determined at a single time point so we cannot exclude a certain variability of fibrin parameters over time. Third, experiments on purified fibrinogen were not performed. They could have helped to assess a contribution of posttranslational fibrinogen modification to clot abnormalities reported here. The precise molecular mechanisms underlying abnormal clot phenotype in HMB remain to be elucidated. We cannot exclude that co-existence of thrombophilia might modify the bleeding risk and fibrin clot phenotype in this clinical setting [9,30]. Fourth, the measurement of fibrinopeptide A cleavage would be useful in evaluating slower fibrin formation in HMB patients. This test has not been performed in the current project. Finally, in clinical practice, like in our study, assessment of abnormal menstrual bleeding is largely based on the patient’s experience and could be imprecise like data on other bleeding manifestations. Moreover, several studies have shown that a mere measurement of blood loss does not precisely characterize all clinical aspects of excessive menstruation [31–33].

In conclusion, the current study shows that increased clot permeability and susceptibility to lysis may represent novel characteristics of women aged below 50 years who suffer from HMB without known organic or hemostatic abnormalities. Our observations suggest that a hemorrhagic clot phenotype can be found among subjects with idiopathic bleeding tendency. Larger groups of patients with HMB are needed to validate our observations and their practical implications.
